# An integrated dataset on current adoption practices, readiness and willingness to use m-commerce amongst women fish vendors in Karnataka state, India

**DOI:** 10.1016/j.dib.2019.103887

**Published:** 2019-04-01

**Authors:** Raghavendra Prabhu, Vasanth Kamath, Harisha Joshi

**Affiliations:** a Manipal Academy of Higher Education, India; b T A Pai Management Institute, India

**Keywords:** m-Commerce, Fisheries, Entry barriers, Economic development

## Abstract

The dataset describes the survey data on current adoption practices, readiness and willingness amongst woman fish vendors (WFVs) towards the adoption of m-commerce in Coastal Karnataka region, India. The coastal Karnataka belt houses three districts viz. Udupi, Uttara Kannada and Dakshina Kannada. Primary data is retrieved from 383 WFVs who serve 26 markets in the districts mentioned above. The dataset comprises of demographic details of the respondents like age, education, per-day sales etc. and specific responses to the items measuring their current adoption practices, as well as readiness and willingness to use m-commerce in their day to day business activities. Availability of this data will help researchers, policy makers and social entrepreneurs to determine optimal strategies towards efficient adoption of m-commerce models amongst small vendors in similar emerging markets thereby increasing their income levels.

**Table:** Specifications Table

Subject area	Social Sciences, Agricultural Sciences, Economics
More specific subject area	Decision making towards enhancing the quality of life amongst WFVs.
Type of data	Table
How data was acquired	Questionnaire-based personal interview based on random sampling.
Data format	Analysed
Experimental factors	Current practices, readiness and willingness among WFVs to adopt mobile commerce.
Experimental features	383 respondents were chosen based on simple random sampling approach across 3 major ports in coastal Karnataka region.
Data source location	Ports in Udupi, and Dakshina Kannada district. Major markets of Uttara Kannada districts in Karnataka, India
Data accessibility	" Data in determinants to use m-commerce among fisherwoman retailers in India”, *Mendeley Data*, v1, https://doi.org/10.17632/7fbn7cdjnv.1
Related research article	R. Prabhu and H. Joshi, Determinants of Willingness to Adopt M-Commerce among Fisher Women Retailers in Karnataka, India, AGRIS on-line Papers in Economics and Informatics, 10(4) (2018), pp. 59–64, DOI: https://doi.org/10.7160/aol.2018.100406[1].

**Table:** 

**Value of the data** • The datasets provide an insight into the m-commerce adoption practices amongst WFVs [1] in the context of emerging economies. • Open access publication of this dataset has an inherent ability to be used by public policy makers or to gain an understanding of the industry practices. • Correctly, the dataset can be used towards creating awareness of the benefits of m-Commerce on marketing decisions [2], [3], providing training [4] and building business models [5], [6] to improve the quality of life, specifically in the context of WFVs in emerging economies.

## 1 Data

**Fig. 1:**
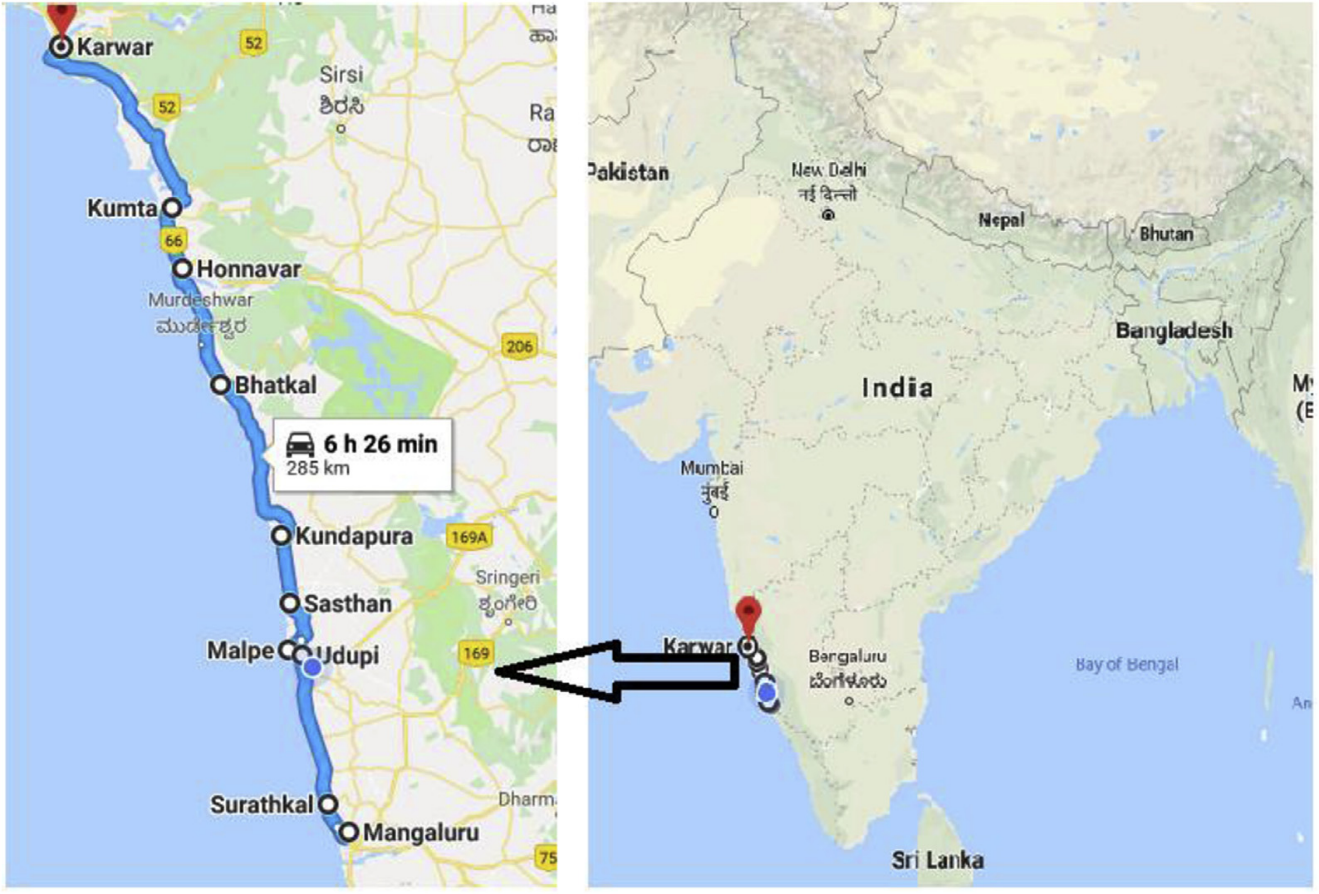
The coastal stretch of Karnataka Source: Google Maps.

This dataset provides information about current adoption practices, readiness and willingness to adopt m-commerce amongst WFVs of coastal Karnataka region, India (Fig. 1). The coastal range of Karnataka spans around 285 Kms from the city of Mangalore in the south to Karwar in the north.

The dataset contains information about the perceptions of the WFVs regarding the m-commerce adoption preparedness. It contains demographic data of the respondents like the age, education levels, and average per day sales. Further it provides data related to the their preparedness towards m-commerce which includes having a bank account for digital transactions, type of mobile phones used by them, knowledge about using SMS services, awareness about cashless payment services, interest in adoption of digital technology enabled services, and finally willingness to use m-commerce.

Data from 383 WFVs are collected and presented along with the survey instrument. Table 1, Table 2 details the description of the each of the parameters and can be used by researchers working in the domain.

**Table 1:** Distribution of fish retailers in 3 districts of coastal Karnataka.

District	Frequency	Percentage
Udupi	166	43.3
Uttara Kannada	132	34.5
Dakshina Kannada	85	22.2
Total	383	100

**Table 2:** Demographic details of the respondents (n = 383).

Variables	Category	Frequency	%
Age	<= 35	58	15.1
	36–45	159	41.6
	46–55	131	34.2
	>55	35	9.1
Education (Class)	Illiterate	56	14.6
	1–3	56	14.6
	4–7	197	51.4
	8–10	68	17.8
	>10	6	1.6
Average/Day Sales(₹)	<= 2500	104	27.2
	2501–5000	171	44.7
	5001–7500	61	15.8
	>7500	47	12.3
Bank Account	Not Present	42	11
	Present	341	89
Bank Type	National	279	72.8
	Cooperative	62	16.2
	NA	42	11
Aadhar-linked	Yes	383	100
	No	0	0
Phone model	No Phone	24	6.3
	Featured	350	91.4
	Smart	9	2.3
Know to use SMS	Yes	82	21.4
	No	301	78.6
Have Debit Card	Yes	282	73.6
	No	101	26.4
Awareness about Cashless Payment	Yes	277	72.3
	No	106	27.7
Customer request for Digital transactions	Yes	65	17
	No	318	83
Interested in digital training	Yes	243	63.4
	No	140	36.6
Willingness to use m-commerce	Yes	229	59.8
	No	154	40.2

In Karnataka, WFVs are generally from the mogaveera^1^ community. They buy small quantities of fish, transport it over short distances, sell it to fixed customers and make minimal earnings. Researchers [7], [8] have studied the role of WFVs in fish sales and their contribution to family income, specifically in the context of coastal Karnataka region. Their role as ‘facilitators’ of fish sales and distribution is truly noteworthy. Although WFVs form only a small section of the total fish trade, they are a vital link between suppliers and consumers.

**Fig. 2:**
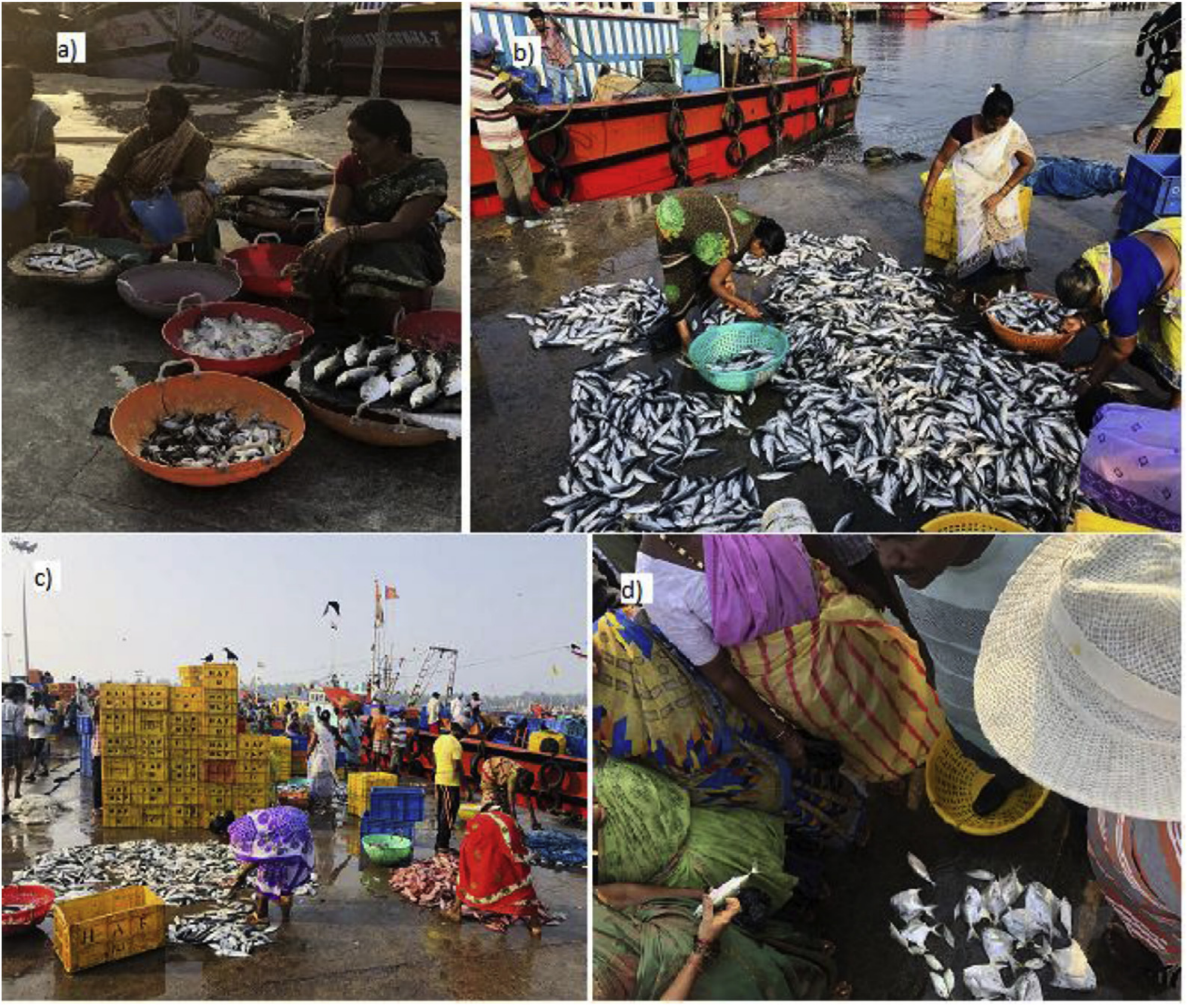
WFVs in various fish trade activities at Malpe harbor. a) Selling of fish; b and c) Sorting of fish; d) Auctioning of the fish.

On any typical day, the WFVs usually participate in auctions, purchase fish subject to their capacity to sell within the same day, their relative negotiating position in the market being served, and limited by the level of consumer demand (Fig. 2). While wholesalers buy large quantities to transport over long distances to make high gains, WFVs spend consistently high costs (on transport and ice), make low profits, and spend more physical labour and long work hours.

As majority of the WFVs contribute towards the family income, it becomes important that they are enabled to increase their revenues so that they can contribute effectively. This can only be made possible with a change in their business models by utilizing technology.

Since the past decade, a disruption in mobile phone coverage and usage has been witnessed in India and similar emerging economies. With cheaper data tariffs, the m-commerce has been growing significantly in India. Though m-commerce has been successfully tried and tested in other sectors, little emphasis has been given to the perishable commodity sector, specifically fisheries.

Contemporary literature in m-commerce adoption search led to findings that, the fisheries retail sector is untapped and there exists a significant scope for research leading to the upliftment of fish vendors from social and financial backwardness by adopting technology-based support systems. This dataset from the survey shall help the researchers understand the levels of current adoption practices, and willingness to use mobile phone for their day to day business activity thereby increasing their customer base and profit.

The dataset comprises of survey responses capturing the Socio-demographic characteristics and the perceptions of 383 WFVs relating to the adoption of m-commerce who serve to the 26 markets in Udupi, Uttara Kannada and Dakshina Kannada districts. Table 1 provides the distribution of samples across the districts.

## 2 Experimental design, materials, and methods

As per the 2010 survey [9], 14,867 fish vendors in the coastal Karnataka region, predominantly women who account to 12,382 vendors. Our focus was to collect data from respondents ensuring the right sample size.

Based on the formula presented in the survey site,^2^ a sample size of 373 was arrived at, assuming a 95% confidence level and 5% margin of error. Further [10] suggested that the decision regarding the sample size tends to be based on experience and sound judgment, rather than relying on strict mathematical formulae. Hence, we were encouraged to collect a number that was higher than the mathematically arrived number of 373 respondents.

In this regard, for conducting the field survey, we adopted a random sampling method. Three teams were formulated to visit the point of source in the retail supply chain. The first team collected the data at Mangalore port (Dakshina Kannada district), the second at Malpe port (Udupi district). The teams were stationed at the port exit and were approaching the WFVs for taking part in the survey. As a rule, we approached every 5^th^ WFV walking out from the port exit. The activity took place for six days.

Similarly, the data for Uttara Kannada district was collected at the market premises across major markets in the district. The data collection was done as the WFVs were selectively allowed (based on the communities they belonged to) to enter the port. Care was taken to ensure that the same participants were not approached for the survey.

Altogether, around 814 WFVs were approached. A total of 404 respondents participated in the survey (response rate of 49.63%). Amongst these, 21 responses were discarded as they were incompletely filled. Finally, the data was collected for 383 respondents.

A major challenge faced during data collection was the presence of barriers to participating in the survey, primarily in the form of suspicion. The WFVs were not willing to participate in a survey conducted by a private entity as they feared that was done not keeping the Fisheries Union representatives in the loop.

As a result, we had to approach the Fisheries Union representative and get the survey instrument vetted by them along with a confidentiality clause as far as the individual respondent details are concerned. After a due endorsement from the representatives, the survey was directed among WFVs.

Further, the items in the survey were translated into the local language to get the respondent perspectives. It is observed that the mean age of the respondents was around 45 years (SD = 8.45) with minimum and maximum age of 25 and 75 respectively.

The dataset offers a comprehensive insight into the several characteristics of the WFVs. Table 2 presents the data collected on the WFVs across all three districts.

From the sample, we can observe that, 76% of the respondents are in the age group of 36–55, with a majority in 36–45 (amounting to 159 out of 383 respondents). Hence, it can be inferred that the people belonging to this age group can be easily trained to adopt m-commerce based applications with minimal efforts. Further, it can be observed that, approximately 70% of the respondents have education beyond grade 4. As a result, the people may-not find trouble in understanding the usage of the m-commerce application.

For the adoption of any m-commerce services, it is vital to have a bank account. Data shows that around 89% of the respondents have a bank account of which 72.8% have a nationalised bank account with 100% Aadhar^3^ enrolment. 91.4% of the respondents own a featured mobile phone, and only 2.3% have a smartphone. The reduced usage of smartphone gives an opportunity for social entrepreneurs and policymakers to explore the mobile services for the featured phone through Unified Payments Interface^4^ (UPI) and to create awareness and provide training to use smartphone amongst WFVs. It was very intriguing to see that 63.4% of the respondents were interested in undergoing digital literacy training with 59.8% showing their willingness to use m-commerce for their day to day business.

Finally, the above-mentioned dataset has the potential to reveal several interesting patterns towards adoption of m-commerce amongst WFVs through further research in this area. It is important to ensure that the WFVs are enabled as they contribute significantly towards their family income. In the wake of commodity price rise, and inflation in education and health related costs, it is often challenging to sustain families with the minimal income the WFVs earn. Though this dataset represents the WFVs localized to coastal Karnataka, the trend is similar across the coastline of India, as well as many emerging economies.

## Footnotes

1 http://www.mogaveeracommunity.com/mogaveera-community.html.

2 https://www.surveymonkey.com/mp/sample-size-calculator/.

3 Aadhaar number is a 12-digit Unique ID number issued by the Government of India to the residents [https://uidai.gov.in/].

4 Unified Payments Interface (UPI): a system that powers multiple bank accounts into a single mobile application (of any participating bank), merging several banking features, seamless fund routing & merchant payments into one hood [http://cashlessindia.gov.in/upi.html].
