# Supplementary material for: An integrated dataset on current adoption practices, readiness and willingness to use m-commerce amongst women fish vendors in Karnataka state, India
Source: Data Brief. 2019 Apr 1;24:103887. doi: 10.1016/j.dib.2019.103887 (PMC6468190; doi:10.1016/j.dib.2019.103887)
Supplement: Multimedia component 2 [file mmc2.docx]

**Questionnaire**

Age: _________________________

District: _________________________

Market Place: _________________________

Education Level: ________________________

Average per day Sales: ________________________

Do you have a bank account (Y/N) ___________

If YES, Bank Name ___________________________

What type of Mobile you use

1. Featured Phone 2. Smartphone 3. I don’t have Mobile

Do you know to operate SMS Service (Y/N) __________

Do you have Aaddhar Card (Y/N) ____________

Do you have Debit Card(Y/N) ______________

Have you used Mobile Banking services(Y/N) _______________

Are you aware of cashless payment(Y/N) ____________

Has any customer requested for digital transaction while conducting business (Y/N) ____________

Are you interested in undergo training in digital payment services(Y/N) ___________

Are you willing to use m-commerce in your day to day business(Y/N) __________
